# Critical Slowing Down Governs the Transition to Neuron Spiking

**DOI:** 10.1371/journal.pcbi.1004097

**Published:** 2015-02-23

**Authors:** Christian Meisel, Andreas Klaus, Christian Kuehn, Dietmar Plenz

**Affiliations:** 1 Section on Critical Brain Dynamics, National Institute of Mental Health, Bethesda, Maryland, United States of America; 2 Department of Neurology, University Clinic Carl Gustav Carus, Dresden, Germany; 3 Institute for Analysis and Scientific Computing, Vienna University of Technology, Vienna, Austria; École Normale Supérieure, College de France, CNRS, FRANCE

## Abstract

Many complex systems have been found to exhibit critical transitions, or so-called tipping points, which are sudden changes to a qualitatively different system state. These changes can profoundly impact the functioning of a system ranging from controlled state switching to a catastrophic break-down; signals that predict critical transitions are therefore highly desirable. To this end, research efforts have focused on utilizing qualitative changes in markers related to a system’s tendency to recover more slowly from a perturbation the closer it gets to the transition—a phenomenon called critical slowing down. The recently studied scaling of critical slowing down offers a refined path to understand critical transitions: to identify the transition mechanism and improve transition prediction using scaling laws.

Here, we outline and apply this strategy for the first time in a real-world system by studying the transition to spiking in neurons of the mammalian cortex. The dynamical system approach has identified two robust mechanisms for the transition from subthreshold activity to spiking, saddle-node and Hopf bifurcation. Although theory provides precise predictions on signatures of critical slowing down near the bifurcation to spiking, quantitative experimental evidence has been lacking. Using whole-cell patch-clamp recordings from pyramidal neurons and fast-spiking interneurons, we show that 1) the transition to spiking dynamically corresponds to a critical transition exhibiting slowing down, 2) the scaling laws suggest a saddle-node bifurcation governing slowing down, and 3) these precise scaling laws can be used to predict the bifurcation point from a limited window of observation. To our knowledge this is the first report of scaling laws of critical slowing down in an experiment. They present a missing link for a broad class of neuroscience modeling and suggest improved estimation of tipping points by incorporating scaling laws of critical slowing down as a strategy applicable to other complex systems.

## Author Summary

Neurons efficiently convey information by being able to switch rapidly between two different states: quiescence and spiking. Such sudden shifts to a qualitatively different state are observed in many complex systems; the often dramatic consequences of these tipping points for diverse fields such as economics, ecology, and the brain have spurred interest to better understand their transition mechanisms and predict their sudden occurrences. By studying the transition from neuronal quiescence to spiking, we show that the quantitative scaling laws for critical slowing down, i.e., a system’s tendency to recover more slowly from perturbations upon approaching its transition point, inform about the underlying bifurcation mechanism and can be used to improve the prediction of a system’s tipping point.

## Introduction

Rapid transitions to a qualitatively different state can be observed in many complex systems. Their sometimes catastrophic outcomes in systems from diverse fields such as climate, ecology, medicine and economics have led to an increased interest in the underlying structure and dynamics of these transitions [1, 2]. While the consequences of such shifts are often undesired, the proximity to a transition can also have various beneficial aspects such as to allow for rapid switching between different states and for small changes to have a large effect on the system state. In the brain, for example, this double-edged role is illustrated by the unwanted transition from normal to epileptic brain activity on one side [3, 4], and, on the other side, the role state transitions play in changing between mutually exclusive motor programs [5] or the generation of action potentials to efficiently convey information.

Better insight into these transitions has come from a dynamical systems’ perspective. For individual neurons, this approach identified two robust mechanisms for the transition from subthreshold near-steady activity to repetitive spiking, saddle-node and Hopf bifurcation [6–9]. The type of threshold behavior predicted by these bifurcations has been able to account for various observations in biological neurons. For example, the smooth frequency vs. current (f-I) curve observed in pyramidal neurons stimulated with steady current is predicted by a saddle-node on invariant cycle bifurcation [8, 10]. Conversely, a discontinuous f-I curve characterized by an abrupt onset of firing as current injection is ramped up has been discussed in the context of an underlying (subcritical) Hopf bifurcation [7, 10, 11]. Although the above mapping between bifurcation and continuity of the f-I curve is not perfect [7] and the experimental determination of the underlying bifurcation to spiking can be problematic [12–14], these differences in f-I curves have led to a classification of neurons according to type 1 and type 2 behavior referring to continuous and discontinuous f-I curves, respectively. However, other crucial predictions following from theory of bifurcations have not been demonstrated experimentally. In particular, theory implies that system dynamics should recover more slowly from small perturbations upon approaching the bifurcation or tipping point, a phenomenon called critical slowing down [15]. Critical slowing down can be monitored by measuring the recovery rate of system variables after small perturbations but also manifests itself by an increase in its fluctuations, i.e. variance due to the longer relaxation times near the bifurcation, as well as higher autocorrelation values [16, 17]. Although theory provides precise quantitative predictions on signatures of critical slowing down for different bifurcations, direct experimental evidence in neurons approaching their spiking threshold has been lacking. The confirmation of critical slowing down and its characteristic scaling in biological neurons therefore represents a missing link between experiment and theory [18] relevant for a large class of neuroscience modeling.

Critical slowing down has furthermore attracted considerable attention in a wide range of systems outside of neuroscience. In many real-world settings, warning signals of impending critical transitions are highly desirable because it is often difficult to revert a system to the previous state once a critical transition has occurred [1, 19]. While qualitative changes in markers related to slowing down have previously been used to probe the proximity to a tipping point in various systems [20–24], a quantification of their scaling laws, to our knowledge, has never been attempted in an experimental setting. Consequently, the affirmation of scaling laws of critical slowing down in a real-world system could offer refined approaches to the prediction of tipping points by incorporating knowledge about these scaling relations.

In the present work, we quantitatively study the scaling laws of critical slowing down for the transition from quiescence to spiking in cortical neurons recorded in the acute brain slice. We show that this transition equates a critical transition exhibiting slowing down where changes in variance and recovery rate are necessary consequences when the bifurcation point is approached. Using bifurcation theory we derive the precise scaling laws relevant in the context of neuronal spiking and compare them to the scaling of variance and recovery rate observed in biological neurons. Our analysis suggests the scaling of these markers of critical slowing down to be governed by a saddle-node bifurcation in both type 1 and type 2 neurons. Furthermore, incorporation of these scaling laws improves bifurcation point prediction from a limited window of observation. To our knowledge this work represents the first quantitative analysis of scaling laws governing critical slowing down in a real-world experimental system.

## Results

### Critical slowing down near the transition to spiking in biological neurons

**Fig 1:**
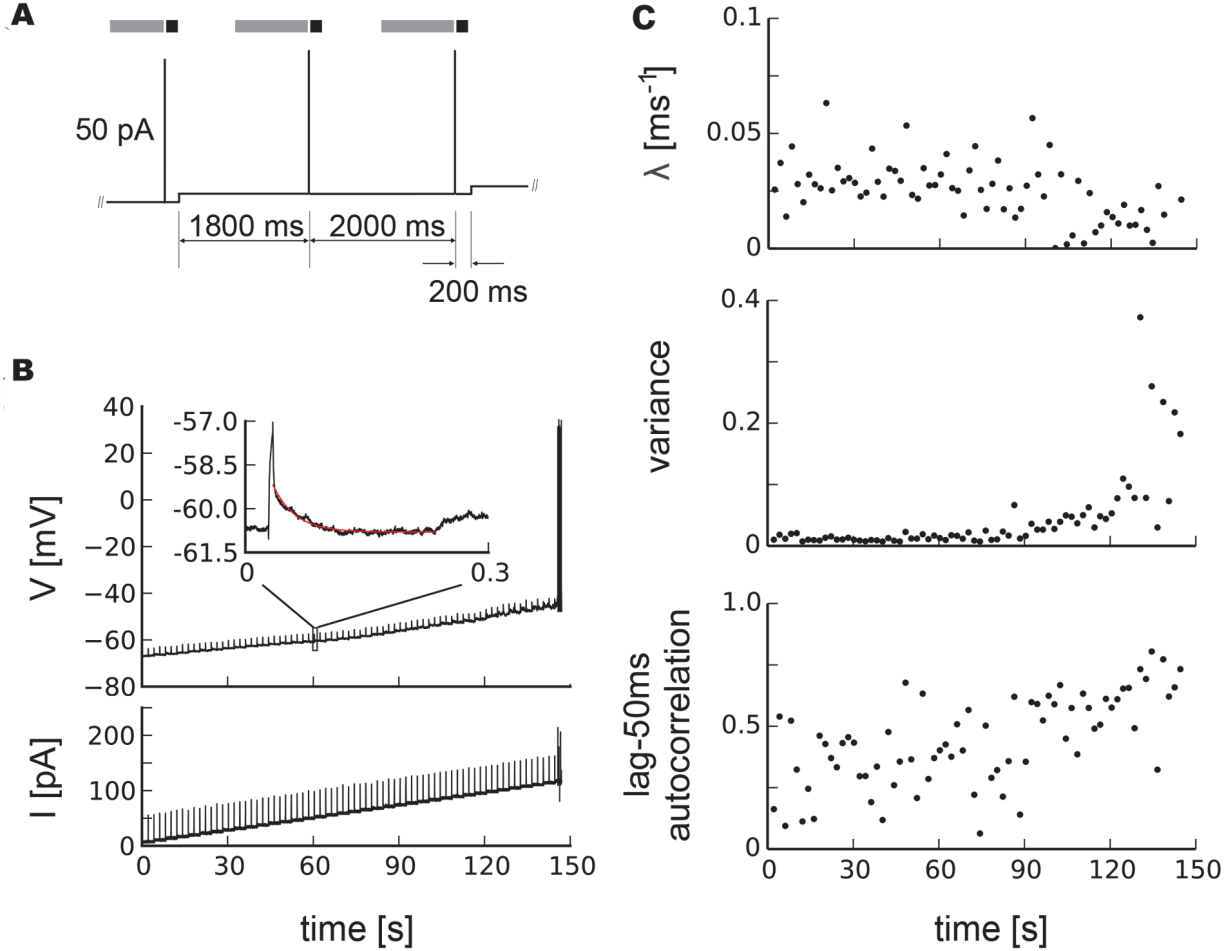
Critical slowing down before neuronal spiking in a pyramidal neuron. a, Current stimulation protocol, gray areas mark segments from which variance and autocorrelation were calculated, black areas segments used to determine recovery rates. b, Time course of the membrane potential subject to brief perturbations by current injections on top of a slowly depolarizing step current. The inset shows a magnification of the voltage response to a short current injection and an exponential fit to its recovery (red line). c, Recovery rates *λ* after perturbations, variance and lag-50ms autocorrelation in the subthreshold voltage, in this case for a pyramidal neuron.

Using the whole-cell patch configuration and acute slices prepared from 2–4 week old rats, we recorded intracellularly the membrane potential of cortical pyramidal neurons and fast-spiking interneurons in response to current injections. We developed a stimulation protocol that allowed us to monitor markers of critical slowing down [1, 2, 16] while systematically increasing the injected current to drive the neuron towards its spiking threshold, i.e. tipping point (Fig. 1 a, b). Specifically, while GABAergic and glutamatergic synaptic transmission was blocked by bath application of PTX (50*μM*) and AP5/DNQX (50*μM*/10*μM*), respectively, we applied a slowly depolarizing step current to gradually drive the membrane potential towards the spiking threshold. In addition to the step current, we applied brief, subthreshold current pulses at regular intervals as small perturbations. When the neuron started to spike the current injection was stopped. We quantified the neuron’s recovery to each perturbation by fitting an exponential decay to the return of the membrane potential trajectory within a few hundred milliseconds to derive a recovery rate *λ* (Fig. 1 b). The unperturbed one—second segments before the current pulses were used to calculate variance and autocorrelation from subthreshold voltage. Starting at the resting membrane potential, recovery rates exhibited a gradual decline which became more pronounced towards the spiking threshold. Concomitantly, autocorrelation and variance showed a marked increase towards the membrane potential value at which spiking started (Fig. 1 c). The decrease in recovery rate together with the increase in variance and autocorrelation amount to conclusive evidence for critical slowing down in subthreshold neuronal activity prior to spiking.

The existence of critical slowing down is a direct consequence of a bifurcation underlying the transition from quiescence to spiking in neurons [6, 7, 25, 26]. Although there have been experimental reports of changes in subthreshold activity dependent on the level of depolarization in cortical neurons, such as the width and decay of exitatory postsynaptic potentials (EPSPs) [12, 27, 28] or the amount of subthreshold voltage noise [29], these observations have not been put into context with critical slowing down at a bifurcation. Even more so, there has been no quantification of these phenomena, which is of particular relevance since bifurcation theory makes precise predictions for the scaling of these markers of critical slowing down. In the following, we will outline in more detail, why it is reasonable to look for critical slowing down in the statistics of subthreshold membrane potential fluctuations near a neuron’s transition from quiescence to spiking. Specifically, we will first present the quantitative scaling laws predicted by theory and second relate them to the scaling observed in experimental measurements from biological neurons.

### Statistical scaling laws of critical slowing down predicted by bifurcation theory

Quiescence or spiking can be regarded as two different states a neuron can be in. The mathematical study of such transitions is called bifurcation theory. From a dynamical systems’ point of view, a neuron’s transition from quiescence to spiking therefore corresponds to a bifurcation in neuron dynamics. It is because of this proximity to a bifurcation that neurons are excitable, i.e., have the ability to exhibit a qualitative change in their dynamics. Neurons can be driven from quiescence toward their spiking threshold by slowly increasing current injections. The observation of a very drastic end of quiescence (or steady-state) upon increase of the injected current beyond a certain threshold suggests that the most likely dynamical transitions are either a saddle-node or a Hopf bifurcation. A saddle-node bifurcation (i.e. a saddle-node bifurcation or a saddle-node bifurcation on an invariant circle bifurcation) is characterized by a single eigenvalue of the linearized subsystem passing through the imaginary axis. A Hopf bifurcation (i.e. a subcritical Andronov-Hopf bifurcation) has a complex conjugate pair of eigenvalues passing through the imaginary axis [7, 30–32]. These insights yield the important conclusion that, no matter how one decides to model neurons, one has to analyze the statistics near saddle-node and Hopf bifurcations to obtain the scaling laws near the transition to spiking. Besides being near a local bifurcation, moderate noise levels in the system are a necessary condition to observe critical slowing down, as dynamics could instantly jump to a different attractor when noise levels are too high.

Here, we first review the scaling laws governing subthreshold dynamics for both saddle-node and Hopf bifurcation. These scaling laws for recovery rate and variance are known analytically [33]. We will derive the scaling for recovery rate for saddle-node and Hopf bifurcation relevant for the transition to neuronal spiking from their bifurcation normal forms and numerically illustrate the scaling relations for both bifurcations in a model system that captures our experimental approach.

#### Normal forms

Normal forms are model systems associated with a bifurcation exemplifying the bifurcation type. A normal form of a saddle-node bifurcation is

$$\begin{array}{c} \frac{dV}{dt}=y+V^{2}. \end{array}$$ (1)

There are two equilibria $V=\pm \sqrt{-y}$ for *y* < 0 and the saddle-node bifurcation occurs for *y*_*c*_ = 0. The equilibrium $V^{-}:=-\sqrt{-y}$ is stable since the linearized system around *V* ^−^ is

$$\begin{array}{c} \frac{dX}{dt}=\left(D_{V}f\right)\left(V^{-}\right)X=-2V^{-}X=-2\sqrt{-y}X. \end{array}$$ (2)

Here, *y* is the parameter which controls the distance to the bifurcation. In our patch experiment, this was the difference between the current at which spiking occurred and the current injected. If one assumes *y* quasi-stationary, we may solve (2) and obtain

$$\begin{array}{c} X\left(t\right)=X\left(0\right)e^{-2\sqrt{-y}t}. \end{array}$$ (3)

Equation (3) implies that when dynamics is perturbed slightly away from the stable equilibrium *V* ^−^ for *y* < 0 the trajectory will return to *V* ^−^ exponentially fast and the exponent scales like $𝓞(\sqrt{-y})$ in terms of the *y*-variable as *y*↗0. Consequently, the closer one gets to the bifurcation point *y*_*c*_ = 0, the longer it takes to recover from a perturbation which is the well-known phenomenon of critical slowing down or intermittency [1, 15, 34]. The factor $-2\sqrt{-y}$ is also refered to as the recovery rate *λ* [33] or as the first Lyapunov exponent [35].

For the Hopf bifurcation the scaling law for recovery rate can be derived in a similarly straightforward fashion. The normal form of the (subcritical) Hopf bifurcation is given by

$$\begin{array}{ccc} \frac{dV_{1}}{dt} & = & yV_{1}-V_{2}+V_{1}\left(V_{1}^{2}+V_{2}^{2}\right), \end{array}$$ (4)

$$\begin{array}{ccc} \frac{dV_{2}}{dt} & = & V_{1}+yV_{2}+V_{2}\left(V_{1}^{2}+V_{2}^{2}\right). \end{array}$$ (5)

The equilibrium *V*_1_ = 0 = *V*_2_ is stable for *y* < 0 and the Hopf bifurcation occurs at *y*_*c*_ = 0. Linearizing around the equilibrium yields

$$\begin{array}{ccc} \frac{dX_{1}}{dt} & = & yX_{1}-X_{2}, \end{array}$$ (6)

$$\begin{array}{ccc} \frac{dX_{2}}{dt} & = & X_{1}+yX_{2}, \end{array}$$ (7)

so that the recovery rate scales with 𝓞(y) as *y*↗0 for the Hopf bifurcation.

The same scaling laws for recovery rate also hold when moderate noise levels are included as is often the case in biological systems. Furthermore, the inclusion of noise terms in the normal forms allows the derivation of scaling laws for variance as a function of the distance to the bifurcation. These scaling laws for recovery rate *λ* and variance *v* under noise conditions have been analytically derived elsewhere and can be summarized, under suitable technical assumptions [36, 37], by

$$\begin{array}{c} \begin{array}{ccc} \lambda & \sim & {\left(-y\right)}^{0.5},\\ v & \sim & {\left(-y\right)}^{-0.5} \end{array} \end{array}$$ (8)

for saddle-node, and

$$\begin{array}{c} \begin{array}{ccc} \lambda & \sim & {\left(-y\right)}^{1.0},\\ v & \sim & {\left(-y\right)}^{-1.0} \end{array} \end{array}$$ (9)

for Hopf bifurcation as *y*↗0 = *y*_*c*_, where *y*_*c*_ is the bifurcation point.

#### Scaling at saddle-node bifurcation and Hopf bifurcation in a model system

As an illustration of the scaling laws governing critical slowing down we modeled the dynamics near saddle-node and Hopf bifurcation in a way that directly relates to our experimental work. Specifically, we were interested in the scaling of variance and recovery rate near these bifurcations. Since we are mainly interested in the scaling of critical slowing down near the transition to continuous spiking, whose dynamics can be shown to be determined by the local bifurcation in question [7, 38], we study the scaling using normal forms of saddle-node and Hopf bifurcation, respectively. Furthermore, the focus on normal forms allows our results to be applied to other systems besides neuron models. For the study of these scaling laws in specific neuron models we refer the reader to [18].

We modeled the saddle-node bifurcation by

$$\begin{array}{c} \begin{array}{ccc} dV & = & \left(-y+\rho V^{2}\right)\;dt+\sigma_{1}\;dW_{1}+\sigma_{2},\\ dy & = & -\epsilon \;dt. \end{array} \end{array}$$ (10)

**Fig 2:**
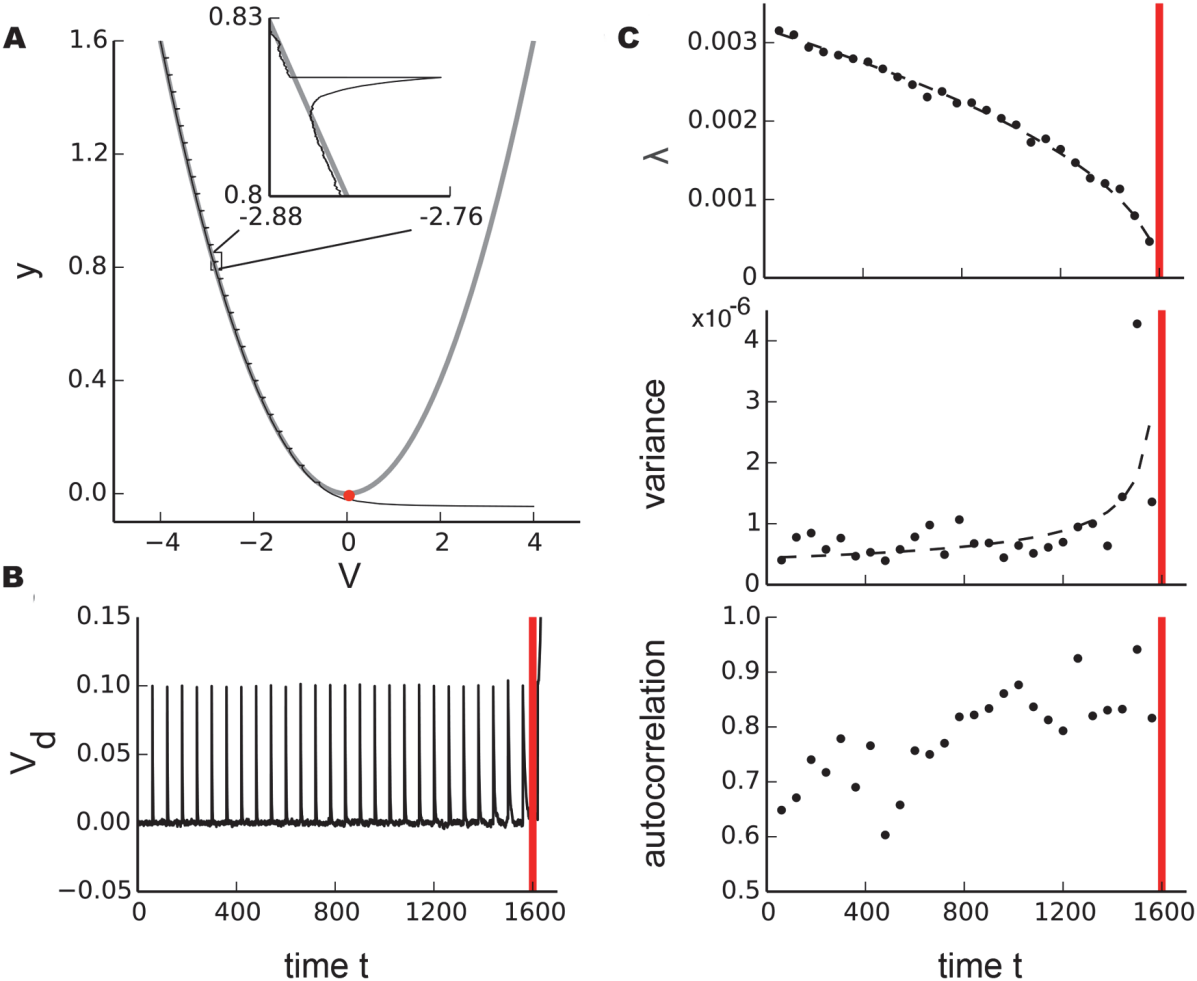
Illustration of stochastic scaling laws near the saddle-node (fold) bifurcation in a model system. a, Phase space with a single stochastic sample path (black) of a saddle-node bifurcation (eq. 10) for the initial condition (*V*(0), *y*(0)) = (−4, 1.6) with *σ*_1_ = 0.001, *ϵ* = 0.001 and small perturbations of size *σ*_2_(*t*_*i*_) = 0.1 with *t*_*i*_ = 60. The bifurcation occurs at (*V*_*c*_, *y*_*c*_) = (0, 0) (red dot). The gray curves are the system equilibria (for *ϵ* = 0). b, Sample path *V*_*d*_ plotted as a time series where the equilibrium values have been subtracted (i.e. detrending along the equilibrium branch). c, Scaling of recovery rate *λ*, variance *v* and autocorrelation as dynamics approaches the bifurcation point (red vertical line). Recovery rate and variance follow a power-law scaling with exponents ±0.5 illustrated by black dashed lines.

Note that (10) takes the form of a non-noisy saddle-node normal form for *σ*_1_ = *σ*_2_ = *ε* = 0. *V* can be interpreted as the membrane potential. The second term corresponds to additive white noise of size *σ*_1_ > 0 while the third term *σ*_2_ = *σ*_2_(*t*) desribes small impulsive perturbations onto the dynamics triggered at fixed deterministic time points *t*_*i*_ = 60. The second equation describes the slowly changing control parameter *y* governed by time scale *ε* which drives the system towards the bifurcation point. In the experimental setting, *y* = *I*_*c*_−*I* corresponds to the distance to the critical current at which spiking starts, *ε* is related to the rate by which current is injected into the cell, *σ*_1_ describes the Gaussian noise level in the system and *σ*_2_ the size of small external perturbations in the form of brief injected current pulses (see experimental stimulation protocol for details). In the following we set *ϵ* = 0.001, *ρ* = 0.1, *σ*_1_ = 0.001 and *σ*_2_ = 0.1. Fig. 2 a shows the trajectory of a single stochastic sample path starting from the initial conditions *y*_0_ = 1.6 and *V*_0_ = −4. Here, the bifurcation occurs at (*V*_*c*_, *y*_*c*_) = (0, 0) (red dot) which corresponds to time *t*_*c*_ = (*y*_0_−*y*_*c*_)/*ϵ* = 1600 (red vertical lines in Fig. 2 b, c).

In terms of a neuron’s underlying electrophysiology, the scaling of critical slowing down can be understood by considering the nonmonotonic I-V curve observed at a saddle-node on a limit cycle bifurcation (SNIC) [8, 12, 14, 39]. Such a nonmonotonic curve implies a local maximum above which depolarization activates a net inward current. Until this maximum is reached inputs are integrated. These integration properties are similar to RC circuits. Near the maximum or tangent point, the shape of the steady state I-V curve is approximated as a parabola in the normal form framework (Fig. 2 a). As *I*_*c*_−*I* = *y* is further decreased, there are two branches, a stable (left) and an unstable one (right). The slope of the I-V curve (or normal form parabola) can, in biological terms, be interpreted as the instantaneous conductance *g*. When linearized about the stable branch, as shown above for normal forms, it follows that $g\sim \sqrt{I_{c}-I}$. In integrators such as a RC circuits, the recovery time constant scales with *C*/*g*. Consequently, the recovery rate, being the inverse of the time constant, will scale like $\lambda \sim \sqrt{I_{c}-I}$.

To visualize this scaling, we detrended *V*(*t*) by subtraction of the stable branch and analyzed the time series *V*_*d*_(*t*) (Fig. 2 b) for markers of critical slowing down as dynamics approached the saddle-node bifurcation. Analogously to the experimental protocol, we fit an exponential decay

$$\begin{array}{c} V_{d}\left(t\right)=a∙e^{-\lambda t}+b \end{array}$$ (11)

for the 5000 sample points following each perturbation of size *σ*_2_ to obtain the recovery rate *λ* and calculated variance and autocorrelation (at lag 100) for the 5000 sampling points of the unperturbed membrane potential preceding each perturbation. While the recovery rate decreases, the variance of the unperturbed signal will increase due to the longer relaxation times near the bifurcation. Fig. 2 c illustrates the scaling for recovery rate and variance with exponents ±0.5 as expected for a saddle-node bifurcation as well as the increase in autocorrelation values.

To illustrate the scaling at a Hopf bifurcation we modeled it by

$$\begin{array}{c} \begin{array}{ccc} dV_{1} & = & yV_{1}-V_{2}+V_{1}\left(V_{1}^{2}+V_{2}^{2}\right)\;dt+\sigma_{1}\;dW_{1}+\sigma_{1}\;dW_{2}+\sigma_{3},\\ dV_{2} & = & V_{1}+yV_{2}+V_{2}\left(V_{1}^{2}+V_{2}^{2}\right)\;dt+\sigma_{2}\;dW_{1}+\sigma_{2}\;dW_{2}+\sigma_{3},\\ dy & = & -\epsilon \;dt. \end{array} \end{array}$$ (12)

**Fig 3:**
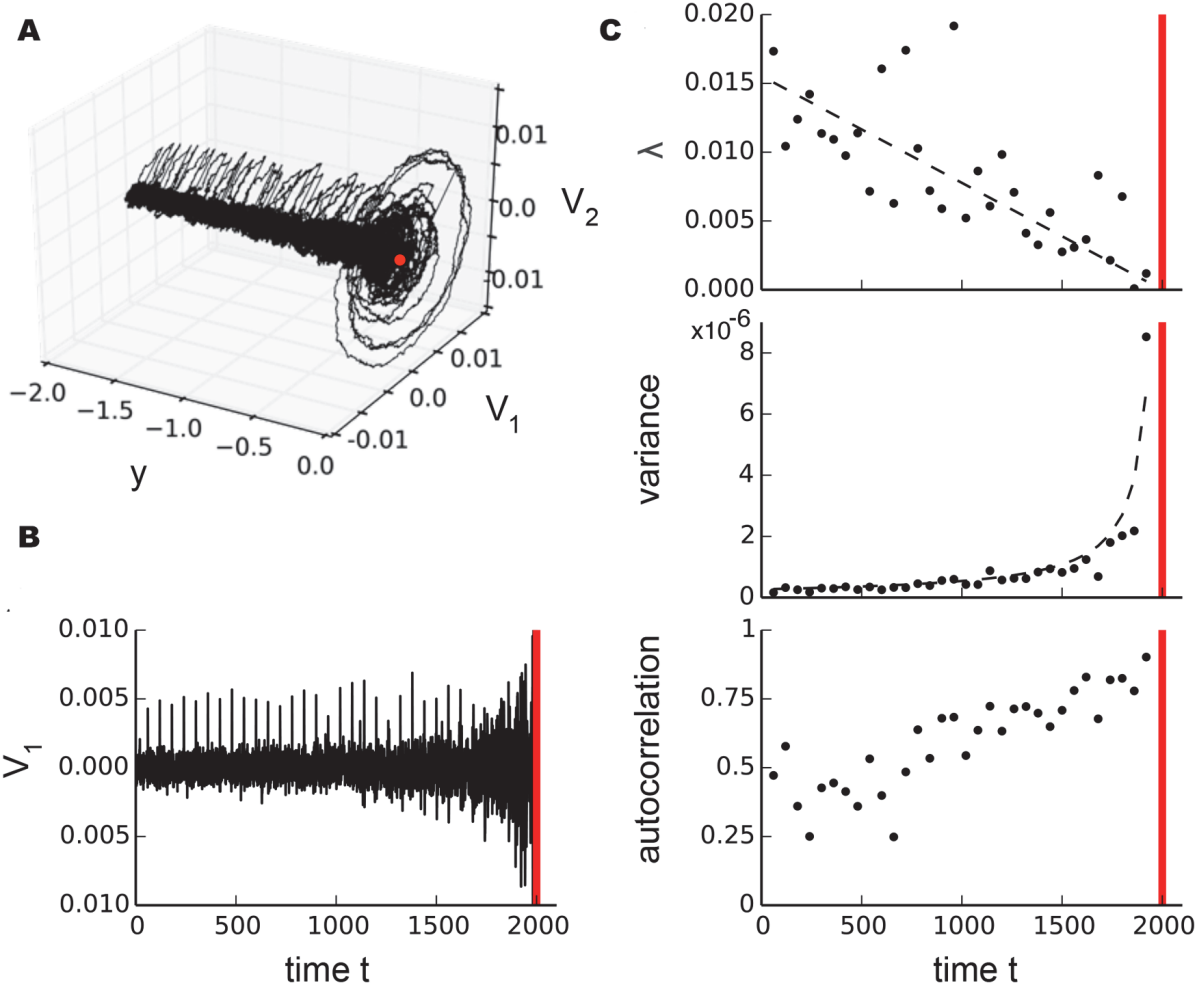
Illustration of stochastic scaling laws near the subcritical Hopf bifurcation in a model system. a, Phase space with a single stochastic sample path (black) of a Hopf bifurcation (eq. 12) for the initial condition (*V*_1_(0), *V*_2_(0), *y*_0_) = (0, 0, −2) with *σ*_1, 2_ = 0.001, *ϵ* = 0.001 and small perturbations of size *σ*_3_(*t*_*i*_) = 0.005 with *t*_*i*_ = 60. The bifurcation occurs at (*V*_1*c*_, *V*_2*c*_, *y*_*c*_) = (0, 0, 0) (red dot). b, Sample path *V*_1_ plotted as a time series used for further analysis. c, Scaling of recovery rate *λ*, variance *v* and autocorrelation as dynamics approaches the bifurcation point (red vertical line). Recovery rate and variance follow a power-law scaling with exponents ±1 illustrated by black dashed lines.

As for the saddle-node bifurcation, (12) takes the form of a non-noisy Hopf normal form for *σ*_1_ = *σ*_2_ = *σ*_3_ = *ϵ* = 0 (eq. (4)). Both *V*_1_ and *V*_2_ exhibit the same scaling behavior, so either one can be interpreted as the membrane potential. Again, white noise of size *σ*_1_ and *σ*_2_ is added and *σ*_3_ desribes the small impulsive perturbations. We set *ϵ* = 0.02, *σ*_1_ = *σ*_2_ = 0.01 and *σ*_3_ = 0.05. Fig. 3 a shows the trajectory of a single stochastic sample path starting from the initial conditions (*V*_1_(0), *V*_2_(0), *y*_0_) = (0, 0, −2). The bifurcation occuring at (*V*_1*c*_, *V*_2*c*_, *y*_*c*_) = (0, 0, 0) (red dot) corresponds to time *t*_*c*_ = 2000 (red vertical lines in Fig. 3 b, c). It has been noted that the membrane near the bifurcation behaves as an underdamped RLC circuit [40]. We derived the recovery rate by fitting the 500 sampling points interval after each perturbation of size *σ*_3_ and variance and autocorrelation from the 5000 sample point segment preceding each perturbation. Fig. 3 c illustrates the scaling for recovery rate and variance with exponents ±−1 as expected for a Hopf bifurcation. This analysis on model systems demonstrates that, in principle, different bifurcation types can be distinguished by the different scaling laws of critical slowing down governing statistics near the tipping point.

### Scaling laws of critical slowing down in biological neurons

We next investigated whether the predicted scaling from theory can be observed in experiment. We thereby focused on neurons for which type 1 and type 2 behaviors have been reported.

#### Pyramidal neurons

**Fig 4:**
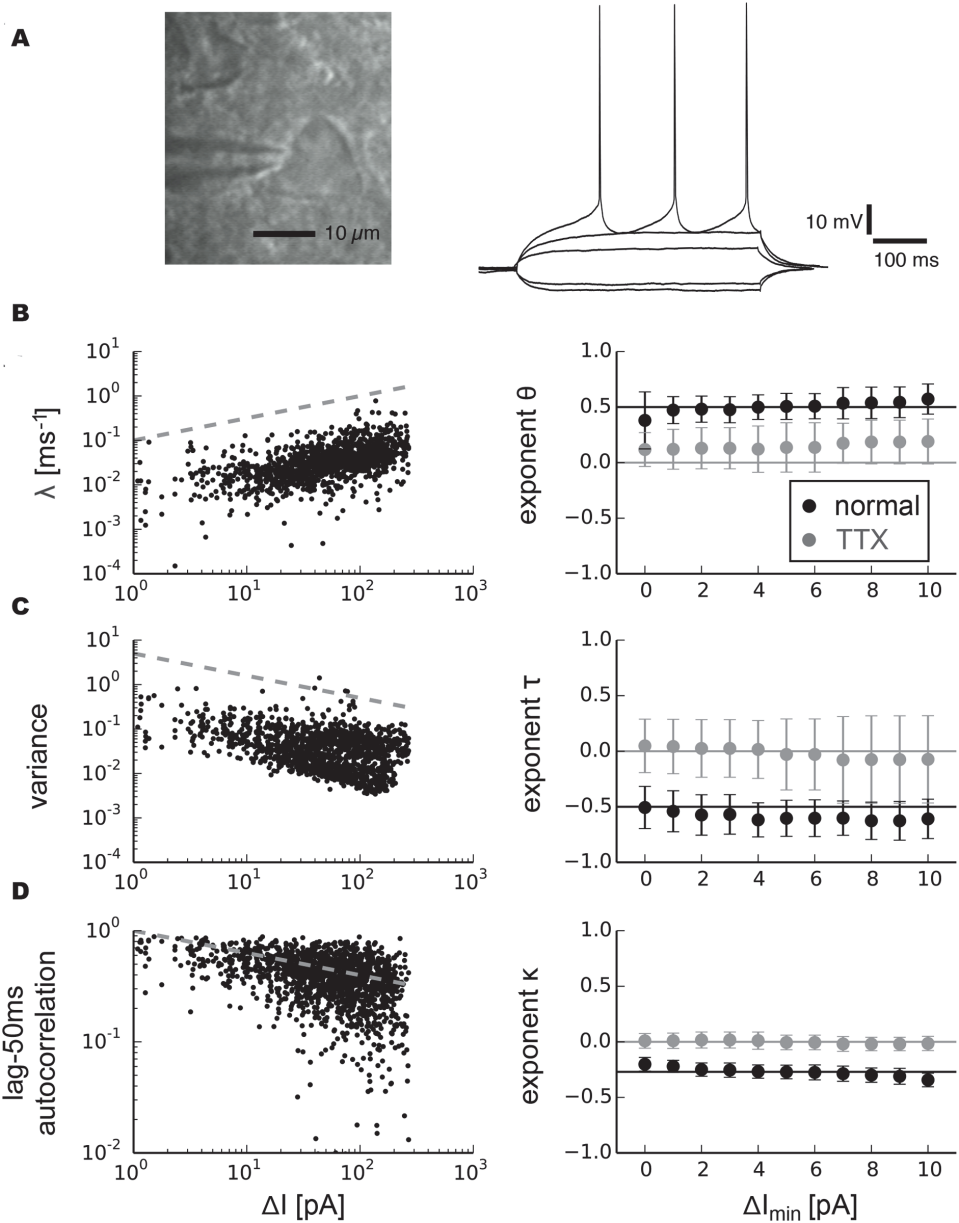
Scaling analysis of indicators related to critical slowing down in pyramidal neurons. a, photomicrograph of a neuron with pyramidal morphology and typical responses to depolarizing and hyperpolarizing currents. b, Recovery rate as a function of Δ*I*, the distance to the bifurcation point, for all trials combined and fitted exponents averaged over individual trials and for different minimal values Δ*I*_*min*_ for normal conditions (right, black markers, standard deviation) and after bath application of tetrodotoxin (right, gray markers, standard deviation). c, Variance. d, Autocorrelation. Grey dashed lines on the left side show power-laws with exponent 0.5 for recovery rate, -0.5 for variance and -0.27 for autocorrelation.

Pyramidal neurons have been suggested to be governed by type 1 behavior under normal conditions [7, 8, 10, 13]. We identified pyramidal neurons by their typical pyramidal morphology (Fig. 4 a), comparatively long-duration action potentials and small afterhyperpolarizations (AHPs). Fig. 4 a shows typical spiking responses of a pyramidal neuron subjected to injected currents with different amplitude levels. In our experimental protocol, we defined the critical current *I*_*c*_ as the average current over the one—second interval prior to the onset of spiking. The distance to the bifurcation in our experimental setup is consequently given by Δ*I* = *I*_*c*_−*I* which corresponds to *y* in our model systems. We fit the recovery rate *λ* for each cell and each trial (n = 9 cells with a total of 22 trials) to Δ*I* by *λ* = *a* ∙ Δ*I* ^*θ*^. To ensure that the derived exponents were independent of the choice of fit intervals, we fit *λ* for a range of data segments with different minimal values Δ*I*_*min*_. The results revealed a *θ* close to 0.5 (Fig. 4 b, black markers, *θ* = 0.50 ± 0.05, mean value ± s.d.). The fitted exponents *τ* for variance robustly centered around a value close to *τ* = −0.5 for sufficiently long segment lengths for which variance was determined (Fig. 4 c, black markers, *τ* = −0.58 ± 0.04). The autocorrelation similarly exhibited a power-law increase for smaller Δ*I* (Fig. 4 d, black markers). The corresponding exponent *κ* depended on the lag for which the autocorrelation was measured.

The transition to spiking in pyramidal neurons occurs within a depolarized voltage range in which many different mechanisms could influence the membrane potential trajectory. In addition to voltage-gated sodium channels, transient A-type potassium channels, low-threshold voltage-gated calcium channels as well as current flow between dentrites and the soma could contribute to the observed change in recovery rate and variance near spike threshold. However, blocking voltage-gated sodium channels in a subgroup of cells (n = 5, 13 trials) by bath application of 1*μM* tetrodotoxin (TTX) completely abrogated spiking at the critical current *I*_*c*_ along with all signatures of critical slowing down (Fig. 4 b–d right side, gray markers). This demonstrated that experimentally observed critical slowing down arises from the basic mechanism that initiates the onset of spiking. Our analysis of critical slowing down in subthreshold statistics yielding exponents ±0.5 is therefore in good agreement with a saddle-node bifurcation underlying the transition to spiking in pyramidal neurons.

#### Fast-spiking interneurons

**Fig 5:**
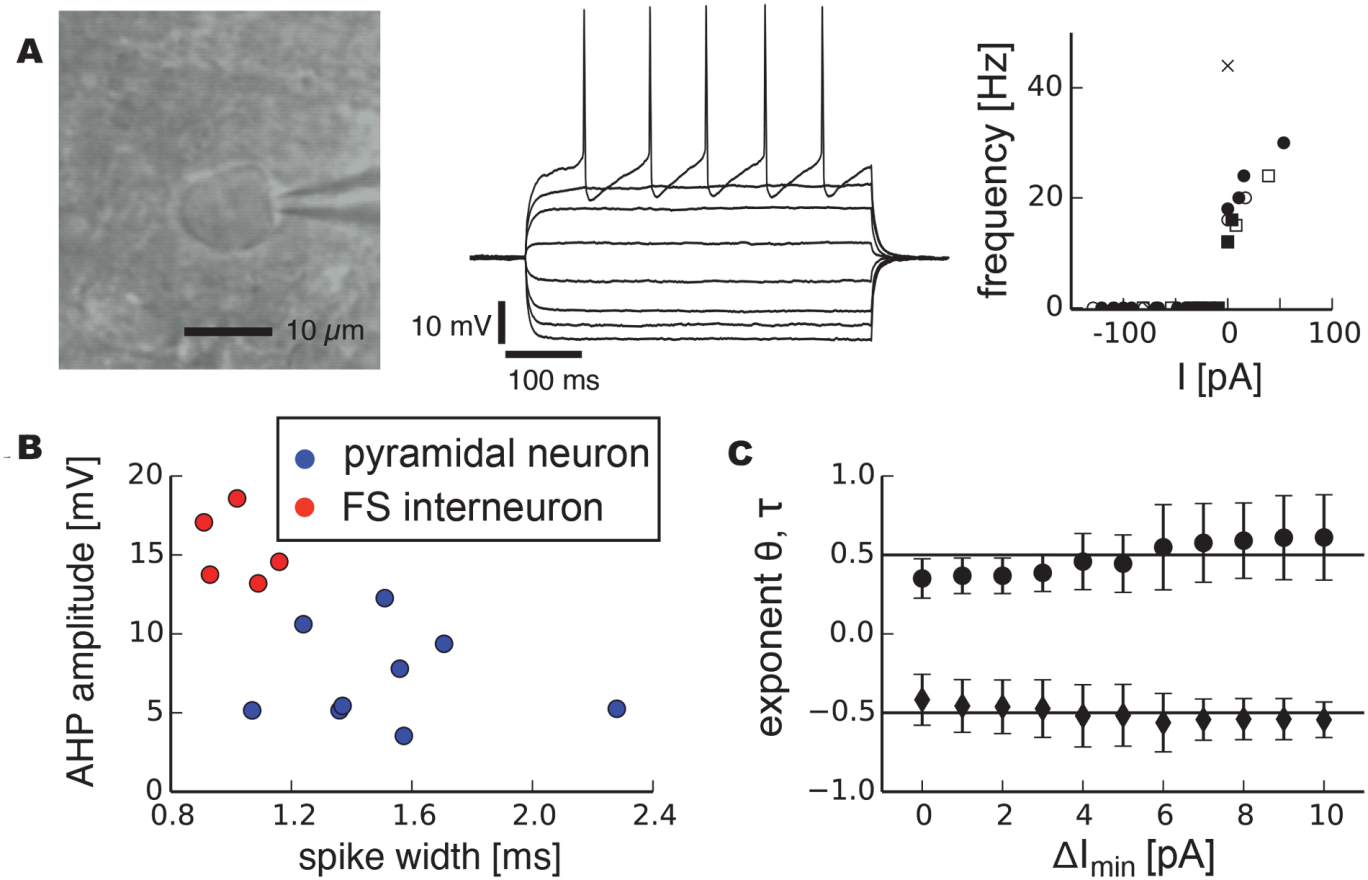
Scaling analysis of indicators related to critical slowing down in fast-spiking (FS) neurons. a, Photomicrograph of a typical FS neuron with round morphology and responses to depolarizing and hyperpolarizing currents. Right: the f-I relationship shows a discontinuity in frequency at the onset of spiking. Different markers correspond to different neurons; for comparability the injected current has been normalized to the onset of spiking. b, FS neurons (red markers) could be distinguished from pyramidal neurons (blue markers) by shorter spike width and greater afterhyperpolarization (AHP) values. c, Exponents (mean ± standard deviation) for recovery rate (*θ*, round markers) and variance (*τ*, diamonds) for different minimal values Δ*I*_*min*_ of the fit.

Type 2 behavior is often assumed to be related to a Hopf bifurcation, although it has been stressed that such a direct mapping is not warranted [7]. To investigate the scaling of critical slowing down in type 2 neurons we extended our analysis to fast-spiking (FS) interneurons. Detailed previous analyses of threshold dynamics in FS interneurons have suggested that these neurons exhibit type 2 behavior characterized by a discontinuous f-I curve [10]. FS interneurons were identified by a nonpyramidal morphology and round soma (Fig. 5 a), short duration of action potentials and strong after-hyperpolarization. Furthermore, when stimulated by current injection we sometimes observed slow oscillations in the membrane potential of FS neurons but never in pyramidal neurons. Fig. 5 a (middle) shows typical spiking responses of a FS interneuron subjected to injected currents with different amplitude levels as well as the discontinuity in f-I curves where the frequency jumps to a relatively high value at the onset of spiking (right). These electrophysiological differences allowed it to reliably distinguish them from pyramidal neurons (Fig. 5 b). In all cells investigated (n = 5, 15 trials) we observed a drecrease in recovery rate and increases in variance and autocorrelation as signatures of critical slowing down upon approaching the spiking threshold. Scaling exponents for recovery rate (*θ* = 0.48 ± 0.10) and variance (*τ* = −0.51 ± 0.05) closely resembled those of a saddle-node bifurcation but not a Hopf bifurcation (Fig. 5 c).

### Prediction of spiking threshold based on critical slowing down

**Fig 6:**
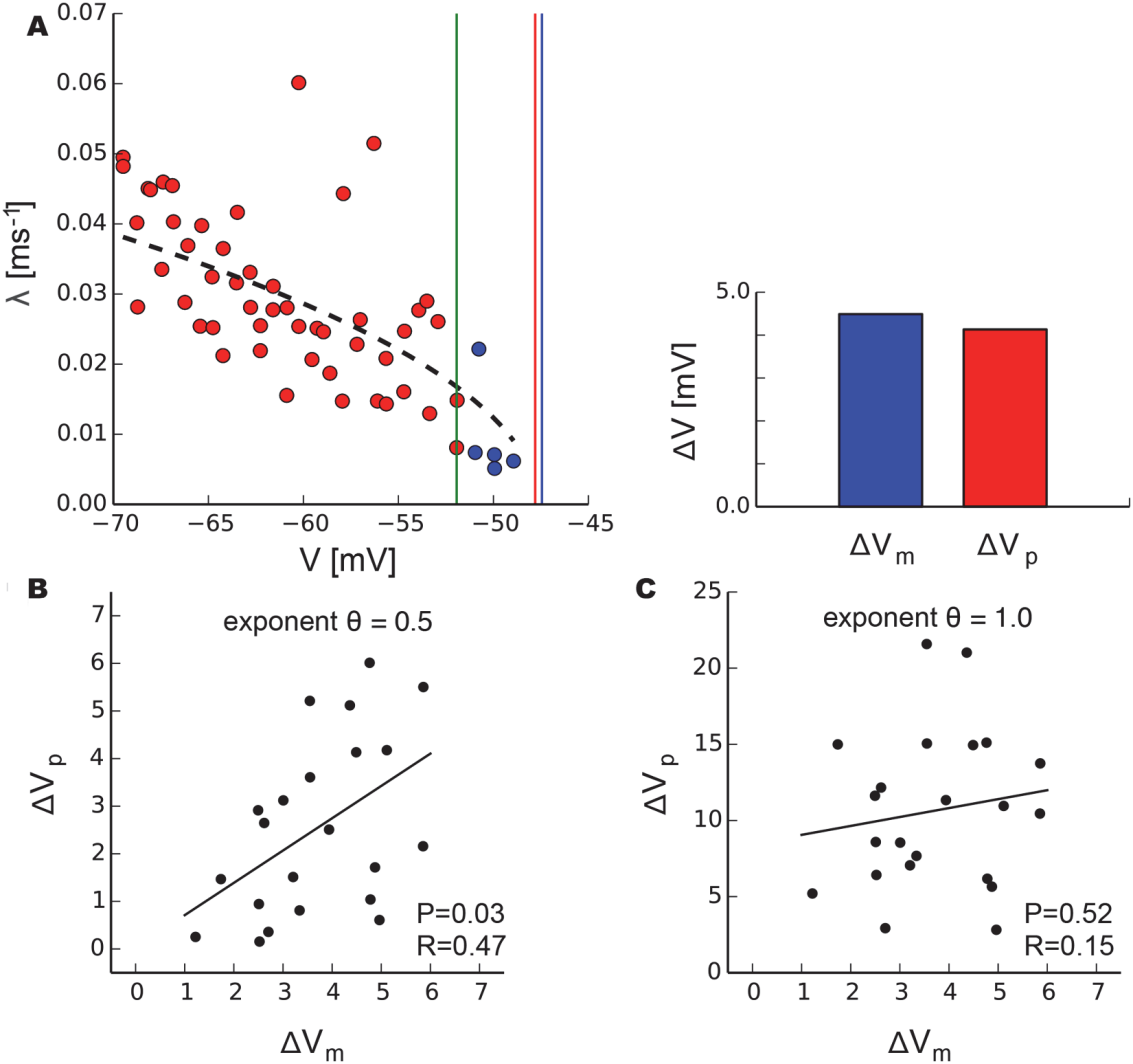
Prediction of the spiking threshold using scaling relations of critical slowing down. a, The critical voltage *V*_*c*_ in pyramidal neurons was determined as a fit parameter by fitting recovery rates *λ* (red markers) excluding the last five measurements (blue markers) to voltage by *λ* = *a*(*V*_*c*_−*V*)^*θ*^. Δ*V*_*p*_ is the difference between the fitted critical voltage (red line) and the last value included in the fit (green line); Δ*V*_*m*_, respectively, refers to the difference between the measured voltage at the onset of spiking (blue line) and the last value used in the fit (green line). b, Predicted Δ*V*_*p*_ and measured Δ*V*_*m*_ exhibit a significant correlation when fitted with exponent *θ* = 0.5 but not when fitted with exponent *θ* = 1.0 (c). P and R values refer to the linear regression analysis (solid black lines).

We hypothesized that the knowledge of the bifurcation that underlies a critical transition could offer a refined approach to predict the bifurcation point from a limited window of observation by using the precise scaling laws governing slowing down. This approach is motivated by many real-world systems exhibiting rare but often catastrophic transitions to a different state. Any insights to better anticipate and predict those transitions would therefore be highly desirable [2]. In particular, it is likely that the control parameter driving the system towards the tipping point, in our case the injected current, may not be directly accessible, but that instead one might be able to monitor some other observable of the system, such as the membrane potential in our experiment. Accordingly, we attempted to predict the voltage $V_{c}^{p}$ at which spiking occurs in pyramidal neurons using only measurements of recovery rate as a function of the neuron’s membrane potential. Omitting the five last data points before spiking (Fig. 6 a, blue markers), we fit *λ* to the membrane potential by $\lambda =a{\left(V_{c}^{p}-V\right)}^{\theta }$ for the remainder of measurements (Fig. 6 a, red markers). This fit allowed the determination of $V_{c}^{p}$ as a fit parameter if *θ* is known (Fig. 6 a, red vertical line). We compared this predicted value to the measured critical membrane potential $V_{c}^{m}$ defined as the average voltage over the one—second interval prior to spiking (Fig. 6 a, blue vertical line) analogous to the definition of *I*_*c*_. The differences between predicted $V_{c}^{p}$ and measured $V_{c}^{m}$ to the last value taken into account for fitting (Fig. 6 a, green vertical line), i.e. Δ*V*_*p*_ and Δ*V*_*m*_, exhibited a significant correlation when data were fit with the saddle-node exponent *θ* = 0.5 (Fig. 6 b). Conversely, there was no significant correlation when prediction was attempted with the exponent *θ* = 1.0 for a Hopf bifurcation (Fig. 6 c). This demonstrates that the knowledge of the underlying bifurcation and its scaling relations for slowing down can be used to estimate the bifurcation point from observation of data.

## Discussion

We showed that the subthreshold membrane potential trajectory in cortical pyramidal neurons and fast-spiking interneurons in the acute slice exhibits critical slowing down indicated by distinct changes in recovery rate, variance and autocorrelation prior to the onset of spiking. To our knowledge, this work is the first to measure and quantify these scaling laws in any experimental setup. The precise scaling of these metrics is in agreement with predictions from bifurcation theory for a saddle-node bifurcation. We demonstrated that incorporation of slowing down scaling laws offers a refined approach to predict the bifurcation point, i.e., spiking threshold in our case, from a limited window of observation. Our findings have implications for neuroscience and, in general, for the understanding of tipping points in complex systems.

### Critical slowing down governed by a saddle-node bifurcation

Numerous experimental studies have demonstrated nonlinear dynamical behavior at the transition to spiking in excitable cells ranging from chaotic attractors and frequency doubling of cardiac pacemaker cells [41] to intermittent bursting in cultured cortical neurons during slow driving [42]. In neurons, the reduction of the spiking mechanism to bifurcations has greatly enhanced the understanding of neuron functioning and is captured by many mathematical neuron models [7]. Although critical slowing down is expected to occur upon approaching the bifurcation point, its actual existence in real neurons had not been rigorously demonstrated. Specifically, the quantification of its scaling laws had been a missing link to theory.

The distinction between type 1 and 2 excitability has proven useful to describe the coding properties of neurons [43, 44] despite the fact that neuronal properties may change on slow time scales for example due to adaptation or bursting [14], cholinergic modulation [13], or changes in the density and distribution of ion channels [45]. A saddle-node bifurcation related to type 1 excitability has been indirectly derived to control spiking in pyramidal neurons from their graded f-I curves [7, 8, 10, 11], their non-monotonic I-V curves, histograms of ISIs, and infinitesimal phase resetting curves [38, 46–48]. The scaling of variance and recovery rate experimentally observed here in pyramidal neurons is well in line with the precise scaling laws predicted by theory for a saddle-node bifurcation.

In FS neurons, whose dynamics at threshold has been described to exhibit type 2 behavior, we also observed scaling with exponents ±0.5. Consequently, these exponents suggest a saddle-node bifurcation instead of a Hopf bifurcation dominating critical slowing down when the spiking threshold is approached from resting membrane potential. Although type 2 excitability is often brought in context with an underyling subcritical Hopf bifurcation, it has been emphasized that this mapping is certainly not clear-cut [7]. A saddle-node (off a stable limit cycle) bifurcation, for example, can result in both type 1 or type 2 excitability and could therefore explain the scaling with exponents ±0.5 observed here. An interesting alternative to a simple saddle-node bifurcation is a folded node which could also underlie the transition to spiking in FS neurons since it generates exponents ±0.5 and can also account for subhreshold oscillations [49] similar to the ones observed here and in [10]. Another possibility in line with exponents ±0.5 would be a singular Hopf, i.e., a mix of fold and Hopf bifurcations whose subthreshold dynamics, however, is governed by the saddle-node [50]. Finally, we should not exclude the possibility that our experimental analysis could provide wrong exponents and that the transition in FS neurons is still governed by a Hopf bifurcation. The fact that the afterhyperpolarization in these neurons is deeper than the fixed point at the previous current level has previously been discussed as one possible indication for a Hopf bifurcation since a similar bistability can be observed in some reduced neuron models, for example [51]. However, given the robust measurement of exponents ±0.5 here and the compatibility of bifurcations exhibiting these exponents with other observations such as subthreshold oscillations as well as the missing definite proof for a Hopf bifurcation in these neurons, it appears more likely that it is rather one of the bifurcations discussed above governing critical slowing down in FS interneurons. In particular when one considers the various factors that can modify a neuron’s bifurcation structure [13, 14, 45] what type of bifurcation actually governs the transition to spiking in a neuron under investigation can only be determined experimentally. The robust observation of scaling laws for slowing down as demonstrated here, is therefore likely to provide informative insights into the composition of an underlying bifurcation structure and can be a useful additional tool in studying the excitability in neurons besides other markers such as the I-V curve, histograms of ISIs and infinitesimal phase resetting curves, for example. Apart from neurons, the different exponents characterizing saddle-node and Hopf bifurcation open the possibility to infer the underlying bifurcation based on subthreshold scaling laws in other systems, too.

The observation of systematic changes in recovery rate and variance as a result of critical slowing down provides a framework to understand previous findings where these metrics were found to be changing depending on the proximity to the spiking threshold. In particular, the width and decay of exitatory postsynaptic potentials (EPSPs) have been observed to be dependent on the level of depolarization in neurons [12, 27, 28] and have been interpreted in the context of changing inward/outward current balances as the membrane potential approaches the spike threshold. In the framework of critical transitions, an incoming EPSP can be understood as a small perturbation to the membrane potential analogously to the brief current pulses in our protocol and will therefore exhibit the same changes in its recovery to baseline. Thus, our results not only are qualitatively in line with the observed broadening of the EPSP shapes observed when approaching the spiking threshold but also provide the distinct quantitative scaling laws by which these changes manifest. Similarly, a positive correlation of the subthreshold voltage noise level to holding potential in pyramidal neurons has previously been observed [29]. This observation links directly to the increase in variance reported here. The scaling laws of variance provide a quantitative framework to describe these previously unexplained observations in the context of critical slowing down.

### Possible implications for information processing in neurons and computational models

The decrease in recovery rate as a result of critical slowing down upon approaching the spiking threshold is likely to have implications on information processing in neurons. It can be expected that changes in the width of EPSPs, analogous to changes in the recovery from small current injections in our protocol, will have an effect on the way by which inputs from other neurons are integrated. The systematic widening of postsynaptic potentials close to spike threshold should progressively facilitate the temporal integration of small inputs to a neuron the closer it gets to the spiking threshold. In this regard, the systematic changes in the form of scaling laws observed in biological neurons can be useful to constrain more realistic computational neuron models. For example, most leaky integrate-and-fire neuron models, by omitting the dynamical modeling of action potential generation, do not take the effects of critical slowing down into account, unless specifically incorporating changes in inward/outward current balance near threshold [52].

One can argue that it might be beneficial for a neuron to balance its excitability in a way that its membrane potential is close to firing threshold allowing for rapid switching between quiescence and spiking at minimal energetic cost. To maintain such a high-conductance state [53], it is conceivable that individual neurons self-organize their excitability [54] and subthreshold statistics such as variance or the length and decay of a transient response such as an EPSP, for example, could consequently be utilized to maintain a neuron close to the spiking threshold. The identical scaling of these statistics in both type 1 and type 2 neurons suggested by our analysis could therefore indicate a universal mechanism by which this tuning towards the bifurcation occurs.

Our finding that both pyramidal neurons and fast spiking interneurons are guided by the same scaling law close to spike onset might have important implications for the balance of fast excitation/inhibition (E/I) in neuronal networks. A precise E/I-balance has been shown experimentally to be maintained in vivo and in vitro as the network undergoes different levels of excitation [55, 56]. Modeling work has demonstrated the E/I-balance to establish a decorrelated network state [57–59]. The I-F curves between pyramidal neurons and fast-spiking interneurons display rather different firing dynamics in response to current pulses. Our work, however, demonstrates that both neuronal populations exhibit similar subthreshold scaling close to spike onset which suggests a symmetrical dynamical regulation of the E/I-balance, which might simplify its maintenance.

### Anticipation of tipping points in complex systems

Beyond single neurons, shifts to different dynamical regimes also occur on a larger spatial scale in neuronal systems. Such transitions of cortical network dynamics can be quite subtle and occur, for example, under physiologic conditions in the course of wake and sleep [60], or are exemplified by the rapid transitions to pathologic seizure states in epilepsy [3, 4]. A tipping point at the network level has also recently been described as ‘coherence potential’ in the ongoing avalanche dynamics of awake monkeys and in vitro [61]. It will be interesting to explore whether these network transitions exhibit similar scaling laws to those reported here for individual cells and whether they could consequently lead to a better understanding and perhaps even prediction of their occurences.

From a more general perspective, our work outlines and applies a strategy of identifying a bifurcation by the scaling relations for markers of slowing down and how to consequently incorporate this knowledge to improve prediction of the transition point. The often irreversible changes that can occur in a large variety of complex systems make signals that warn of these transitions highly desirable [1, 19]. Although in the specific case of neuron firing one might think of alternative approaches to anticipate the onset of spiking such as simple thresholding or assuming an integrate and fire model with a certain amount of noise, these methods may likely not be applicable to other real-world systems. Recently, a particularly promising approach to predict these kind of critical transitions in a large variety of complex systems has been based on variables related to critical slowing down as these can often be readily monitored independently of a system’s specificities. So far, a large body of research work has attended to qualitative changes in markers of slowing down to anticipate tipping points [20–24]. Our work constitutes, to our knowledge, the first experimental system in which the quantitative scaling laws governing slowing down have been reported. We suggest that the refined prediction based on scaling laws demonstrated here could also be applicable to other complex systems. While a direct measurement of recovery rates may not always be feasible, indirect measures such as variance can also be used to infer the underlying transition mechanism. Once an underlying bifurcation has been identified, in principle, the precise scaling laws can be used to predict the tipping point as demonstrated in the current study. Although prediction performance is naturally impeded by stochastic perturbations which can trigger critical transitions even before the bifurcation point is reached [1, 62, 63], we demonstrate that given sufficient data and moderate noise levels, reasonable quantitative predictions become possible. In this respect, our results can be regarded as a proof of concept that an estimation of the proximity to the tipping point based on quantitative scaling of critical slowing down is possible and provide a step forward in estimating the fragility in complex systems.

## Materials and Methods

### Ethics statement

Procedures were in accordance with National Institutes of Health guidelines. Animal procedures were approved by the National Institute of Mental Health Animal Care and Use Committee.

### Preparation of acute slices from rat cortex and whole-cell patch recording

The brains of Sprague Dawley rats (P14-P28) were removed and cut into acute coronal slices of medial prefrontal or somatomotor cortex at 350*μm* thickness (VT1000S, Leica) in ice-cold artificial cerebral spinal fluid (ACSF; 124*mM* *NaCl*, 1.2*mM* *CaCl*_2_, 1*mM* *MgSO*_4_, 3.5*mM* *KCl*, 26.2*mM* *NaHCO*_3_, 0.3*mM* *NaH*_2_ *PO*_4_, and 10*mM* *D*−*Glucose*) bubbled with carbogen (95% *O*_2_, 5% *CO*_2_). All recordings were performed under perfusion flow rate of 3–4*ml*/*min* while continuously monitoring and maintaining temperature at 35±0.5°C. The ACSF’s osmolarity was 290±10*mOsm*. NMDA- and AMPA-mediated synaptic transmission was blocked with bath-application of 50*μM* *AP*5 and 10*μM* *DNQX*, respectively, and GABAa-mediated transmission with 50*μM* *PTX*. Patch pipettes were pulled from borosilicate glass using a P-97 micropipette puller (Sutter Instrument, CA, USA), and had a resistance of 4–9*M*Ω. The intracellular patch solution contained 132*mM* *K*−*Gluconate*, 6*mM* *KCl*, 8*mM* *NaCl*, 10*mM* *HEPES*, 2*mM* *Mg*−*ATP*, 0.39*mM* *Na*−*GTP*, pH adjusted to 7.2–7.4 with KOH. Putative pyramidal or fast-spiking neurons were visualized using an infrared CCD camera (Hamatsu) on a BX50WI (Olympus) upright water immersion microscope. Somatic gigaseals (> 2–4*G*Ω) were made to visually identified cells within superficial layers. After break-through, intracellular membrane potentials were recorded in current-clamp mode (Axopatch 200B, Axon Instruments), pre-amplified and low-pass filtered at 10 kHz (Cyberamp 380, Axon Instruments), and digitized at 25 kHz for voltage and 2.5 kHz for current traces using a CED 1401 (Cambridge Electronic Design). We applied a step current that increased by 3 pA every 4.01 s to slowly drive neurons towards the tipping point at which they would start spiking. On top of this slowly increasing current we induced small perturbations to the membrane potential by injecting current pulses of 50 pA for 5 ms at 1800 ms time and 3805 ms on each step (Fig. 1 a). The recovery after small perturbations allowed to measure recovery rates, the unperturbed segments before current pulses to estimate variance and autocorrelation from subthreshold voltage. Data were collected continuously with Spike2 (CED) and analyzed off-line.

### Data analysis

The recovery rate after each perturbation by 5 ms current injection of 50 pA was determined by fitting the 4800 sample (corresponding to 192 ms at 25000 Hz sampling rate) long segment of subthreshold voltage following the pulse current injection. Prior to fitting, the mean voltage of the segment was subtracted. The recovery of the voltage *V*(*t*) after each perturbation was fit by an exponential decay

$$\begin{array}{c} V\left(t\right)=a∙e^{-\lambda t}+b \end{array}$$ (13)

a,b,λ∈ℝ using the Python (Python Software Foundation, version 2.6) function scipy.optimize.curve fit. For each perturbation, the recovery rate *λ* was then recorded together with the mean voltage over the 1 second interval prior the perturbation and the mean injected current *I* during the 1 second interval prior the perturbation for further analysis. The distance to the bifurcation point, Δ*I*, is then given by Δ*I* = *I*_*c*_−*I* where *I*_*c*_ is defined as the average current injected during the one—second interval before the first spike. Note, that Δ*I* is directly related to *y* in our model systems.

Variance was calculated from subthreshold voltage segments prior to each current pulse. For the results in the main part of the manuscript, segments of one—second duration were taken. Similarly, autocorrelation was calculated from subthreshold voltage segments of one—second duration prior to each current pulse. After subtraction of the mean we derived the autocorrelation function *ACF*(*s*) of a signal *F*_*i*_(*t*) with length *N*, mean *μ* and variance *v* by

$$\begin{array}{c} ACF^{*}\left(s\right)=\frac{\sum_{t=1}^{N-s}\left(F_{i}\left(t\right)-\mu \right)\left(F_{i}\left(t+s\right)-\mu \right)}{v},\;s=1,\ldots ,N/2 \end{array}$$ (14)

and normalization by the first value $ACF(s)=\frac{ACF^{*}(s)}{ACF^{*}(1)}$. For the analysis in the experimental part of the manuscript, we used the value of the autocorrelation function at lag 50 ms.

We determined the power-law exponents governing the scaling for recovery rate, variance and autocorrelation by a linear fit in log-space. Specifically, logarithmic values of recovery rates *λ*, variance *v* and lag-50ms autocorrelation (*ACF*_50*ms*_) were fit individually for each trial as a function of the corresponding logarithmic values of Δ*I* by

$$\begin{array}{c} \log\;x=A∙\log\Delta I+B \end{array}$$ (15)

using the Python function scipy.optimize.curve fit. Here, *x* are the values of recovery rate, variance and autocorrelation, respectively, and *A* corresponds to the related exponent (i.e. *θ*, *τ* or *κ*) obtained in the fit. For the determination of exponents, we required fits to have *R* ^2^ ≥ 0.1. To determine a robust estimate of the exponent the fit values were calculated for different minimal values Δ*I*_*min*_, i.e. Δ*I* values smaller than Δ*I*_*min*_ were discarded in the fit. The exponents given in the main text are the mean values over the different Δ*I*_*min*_.
